# HPV prevalence and genotype distribution in 2,306 patients with cervical squamous cell carcinoma in central and eastern China

**DOI:** 10.3389/fpubh.2023.1225652

**Published:** 2023-08-28

**Authors:** Chunrong Han, Wanqiu Huang, Mei Ye, Rong Zou, Jianyun Lan, Jing Chen, Jingui Jiang, Hongjing Wang, Lin Xia, Jun Luo, Dongbin Li, Jianxiang Geng, Zhihui Wang, Jian Huang

**Affiliations:** ^1^Department of Pathology, Nanjing Lishui People’s Hospital (Zhongda Hospital Lishui Branch), Southeast University, Nanjing, China; ^2^Key Laboratory of Systems Biomedicine (Ministry of Education), Shanghai Centre for Systems Biomedicine, Shanghai Jiao Tong University, Shanghai, China; ^3^Nanjing Jiangning District Maternal and Child Health and Family Planning Service Center, Nanjing, China; ^4^Department of Pathology, Yancheng No.1 People’s Hospital, Yancheng, China; ^5^Department of Pathology, Jingjiang People’s Hospital, Taizhou, China; ^6^Department of Pathology, Jinhu County People’s Hospital, Huai’an, China; ^7^Department of Pathology, Dantu District People’s Hospital of Zhenjiang, Zhenjiang, China; ^8^Department of Pathology, People’s Hospital of Yangzhong City, Yangzhong, China; ^9^Department of Central Laboratory, Jiangsu Health Vocational College, Nanjing, China; ^10^Department of Pathology, Nanjing Meishan Hospital, Nanjing, China; ^11^Department of Pathology, Nanjing Hospital of Traditional Chinese Medicine, Nanjing, China; ^12^Department of Pathology, Linyi Cancer Hospital, Linyi, China; ^13^The Cross-Strait Precision Medicine Association HPV Infection Disease Professional Committee, Nanjing, China

**Keywords:** human papillomavirus, cervical squamous cell carcinoma, high-risk genotypes, prevalence, China

## Abstract

**Background:**

To explore the positivity rate and genotype distribution of human papillomavirus (HPV) in cervical squamous cell carcinoma (CSCC) tissues in central and eastern China and to provide theoretical basis for cervical cancer screening and prophylactic HPV vaccine development in China.

**Methods:**

DNA was extracted from paraffin-embedded tissues of CSCC samples and exfoliated cervical cells of cervical cancer screening populations. 23 HPV genotypes were detected by combining polymerase chain reaction (PCR) and reverse dot hybridized gene chip detection technology in 2,306 CSCC tissues and 10,245 cervical cancer screening populations. The genotype distribution of HPV infection was analyzed.

**Results:**

The overall infection rate of HPVs in 2,306 CSCC patients was 92.71%. The frequency of single-type HPV infection and multiple-type HPV infection were 86.48% and 13.51%, respectively. The most common HPV genotypes detected in Chinese CSCC tissues were HPV-16, HPV-18, HPV-31, HPV-33, HPV-45, HPV-52, HPV-58, and HPV-59. The overall positivity rate of these eight high-risk HPV (HR-HPV) genotypes in HPV-positive CSCC was as high as 96.91%. Of which the positivity rate of seven HR-HPV genotypes related to nine-valent HPV vaccines in HPV-positive CSCC was 95.09%. Meanwhile, the overall infection rates of HR-HPV and low-risk HPV (LR-HPV) in female aged 35–64 years who underwent cervical cancer screening were 13.16% and 1.32%, respectively. The high-frequency HR-HPV genotypes in cervical cancer screening women were HPV-52, HPV-58, HPV-16, HPV-53, HPV-68, HPV-39, HPV-51, and HPV-56, with positivity rates of 2.25%, 1.60%, 1.31%, 1.22%, 0.93%, 0.92%, 0.78%, and 0.74%, respectively.

**Conclusion:**

Among women screened for cervical cancer in China, detecting the 8 high-frequency HR-HPV genotypes can reduce technical difficulty and reagent costs, while also improving the efficiency and effectiveness of cervical cancer screening. HPV genotyping assists gynecologists in assessing the risk of HR-HPV-positive cervical intraepithelial neoplasia and guiding them in implementing appropriate interventions. Furthermore, HPV genotyping is helpful for doctors to follow up HR-HPV-positive women and to evaluate the protective effect of HPV vaccine.

## 1. Introduction

Cervical cancer is the most common malignancy of the female reproductive system and ranks second after breast cancer in developing countries (1). In China, there are approximately 110,000 new cases of cervical cancer and 60,000 deaths each year (2). Human papillomavirus (HPV) infection is closely associated the development of cervical cancer (3). So far, more than 200 different Human papillomavirus (HPV) genotypes have been found in nature (4), of which more than 40 can infect the male and female reproductive tracts, including the skin of the penis, vulva, anus, vagina, cervix, and rectum (4, 5).

These HPV genotypes are further classified into low-risk HPV (LR-HPV) and high-risk HPV (HR-HPV) based on their carcinogenicity. HR-HPVs including HPV-16, -18, -31, -33, -35, -39, -45, -51, -52, -56, -58, -59, are mainly responsible for the development of cervical cancer (6–8). Of which, HPV-16 and HPV-18 are the primary HR-HPV genotypes related to 71% cervical cancer worldwide (9, 10). There are significant differences in the prevalence of different HPV genotypes in cervical cancer (11–13). At present, there is no systematic evaluation of age distribution, HPV genotype distribution and HPV infection rate in patients with cervical squamous cell carcinoma (CSCC) in a large sample in central and eastern China. It is also unclear whether there is significant difference in HPV genotype distribution and infection rate between CSCC patients and cervical cancer screening individuals.

To answer the questions above, we collected tissue samples from 2,306 CSCC patients who underwent surgery, as well as the exfoliated cervical cells from 10,245 cervical cancer screening population. We systematically investigated the prevalence and genotype distribution of HPV in both groups. Our results can provide new clues for further understanding of the pathogenesis of CSCC and the prevention strategies for CSCC.

## 2. Materials and methods

### 2.1. Collection of tissue samples from CSCC patients

From January 2010 to October 2022, we collected 2,306 clinically diagnosed CSCC cases from 48 provincial and county-level hospitals in 30 cities across 6 provinces in central and eastern China (Supplementary Table S1). The patients’ average age was 53 ± 11.89 (ranging from 19–90) years old. All patients underwent radical total hysterectomy, and their surgically excised tissues were pathologically examined. Two experienced pathologists reviewed the slides of paraffin-embedded CSCC tissues and clinical pathological data of the CSCC samples according to the classification criteria of gynecological tumors in WHO (14). This study was approved by the Ethics Committee of Nanjing Traditional Chinese Medicine Hospital (approval no. 2012NJL008).

### 2.2. Specimen collection of exfoliated cervical cells from the healthy women undergoing cervical cancer screening

In this study, a total of 10,245 healthy women were recruited, who aged between 35–64 years old and underwent cervical cancer screening in the community of Jiangning District, Nanjing City, Jiangsu Province in 2022. Exfoliated cervical cells were collected from each participant by experienced professional gynecologists using a plastic sampling brush. The brush was inserted into the cervix and rotated five times in the same direction before slowly being withdrawn. The brush head was then broken off and placed vertically in a labeled sampling tube containing 95% ethanol fixative. The bottle cap was tightly sealed and shaken 5–8 times and stored at 4°C for HPV DNA genotype testing.

### 2.3. DNA extraction from paraffin-embedded tissue and exfoliated cervical cells

Firstly, the excess paraffin around each paraffin-embedded tissue was removed and each paraffin-embedded tissue was cut into 4 μm thick slices, yielding for 3–5 slices. These slices were then placed in a 1.5 mL centrifuge tube and 150 μL lysis buffer was added. Then mix thoroughly and centrifuge instantaneously. The tubes were heated in a metal bath at 100°C for 10 min. Immediately centrifuge at 13,000 rpm for 10 min. The middle layer of the DNA solution was collected for further use. According to the instructions of DNA extraction kit (catalog number DP340, Tiangen biochemical technology Co., Ltd., Beijing, China), the total DNA of exfoliated cervical cells in cervical cancer screening group was extracted.

### 2.4. HPV genotyping test

All DNA samples were tested using the Human Papillomavirus Genotyping kit for 23 Types (HPV 23 full-Genotyping) (catalog number QX202203011) provided by Yaneng Bioscience (Shenzhen) Co., Ltd. This HPV genotyping kit detects 23 genotypes, including six LR-HPV genotypes (HPV-6, -11, -42, -43, -81 and -83) and 17 HR-HPV genotypes (HPV-16, -18, -58, -52, -31, -33, -59, -45, -51, -56, -73, -66, -39, -53, -68, -82, and -35). PCR was conducted on ABI7500 fluorescent detector produced by Life Technology (United States) Co., Ltd. Then, after each specimen was fluorescently labeled by PCR, the presence or absence of hybridization signals at various sites on the membrane chip were then used to determine the results. PCR amplification, hybridization, incubation, and color development were performed according to the instructions.

### 2.5. Statistical analysis

The statistical analysis in this study was conducted using the SPSS 26.0 software (IBM, United States). The data were processed and visualized using GraphPad Prism 8 (GraphPad software, San Diego, United States). Chi-square test was used to analyze the difference in prevalence or attribution between different HPV types. *p* < 0.05 was regarded as statistical significance.

## 3. Results

### 3.1. Introduction of the source of cervical squamous cell carcinoma tissue samples

**Figure 1:**
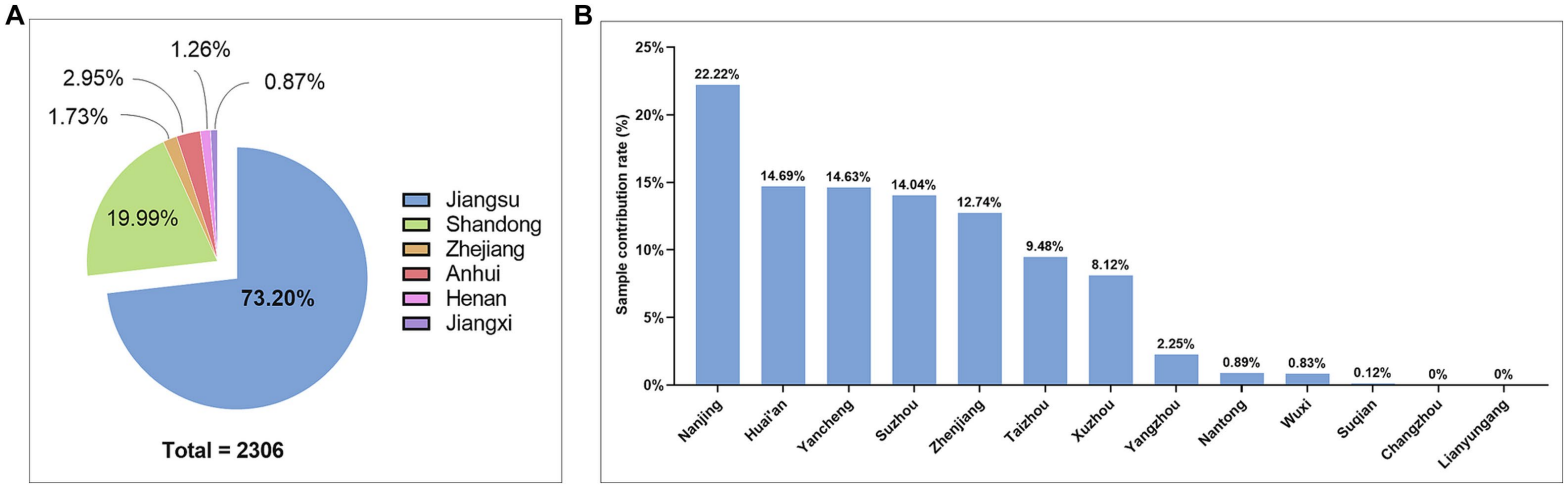
Sample source and geographical distribution in this study. **(A)** Pie chart of sample attribution rate by province; **(B)** Histogram of the sample attribution of by city-level hospitals in Jiangsu Province.

A total of 2,306 cervical squamous cell carcinoma (CSCC) tissue samples were collected from 48 hospitals in six provinces in central and eastern China, including Jiangsu, Anhui, Zhejiang, Henan, Jiangxi, and Shandong. Among them, Jiangsu province contributed the highest number of samples, covering 38 hospitals and 1,688 cases, accounting for 73.20% of the total samples (Figure 1A). The samples from Jiangsu province were collected from 11 prefecture-level cities, of which Nanjing accounted for 22.22%, Huai’an for 14.69%, Yancheng for 14.63%, Suzhou for 14.04%, Zhenjiang for 12.74%, Taizhou for 9.48%, and Xuzhou for 8.12%, respectively (Figure 1B).

### 3.2. Overall prevalence of HPV infection in CSCC patients

**Figure 2:**
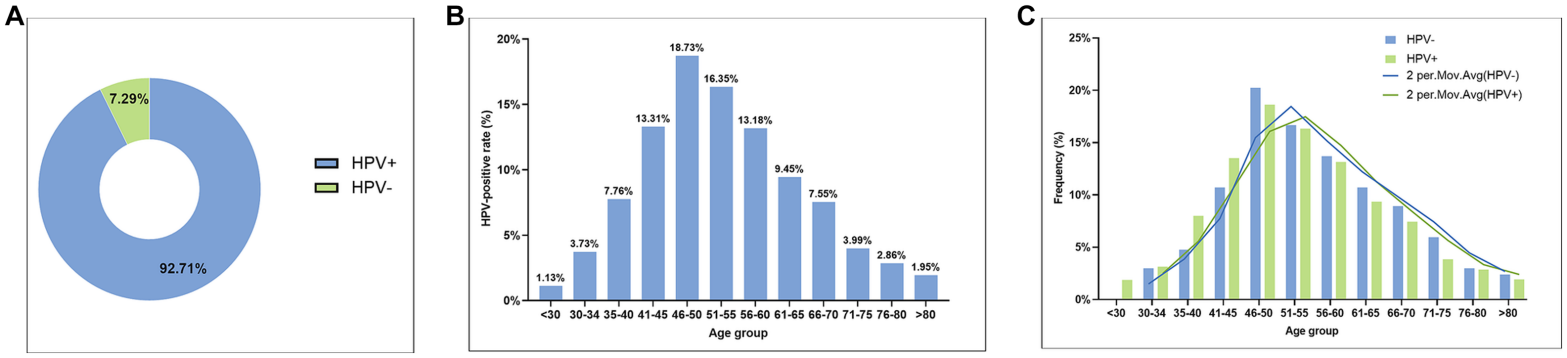
The overall prevalence of HPV infection in CSCC samples. **(A)** Pie chart of HPV-positive and HPV-negative rates in CSCC; **(B)** Histogram of age distribution of HPV infection in HPV-positive CSCC; and **(C)** Comparison of age distribution between HPV-positive CSCC and HPV-negative CSCC.

Among the 2,306 cases of CSCC, a total of 2,138 cases were HPV-positive, accounting for 92.71% (2,138/2,306), while 168 cases were HPV-negative, accounting for 7.29% (168/2.306) (Figure 2A). Among HPV-positive patients, the youngest patient was 19 years old and the oldest was 90 years old. There was an obvious HPV infection peak in the 46–50 age group of HPV-positive CSCC, with a positivity rate of 18.73% (432/2,306) (Figure 2B). Furthermore, patients under the age of 34 accounted for 4.86%, those over 66 years old accounted for 16.35%, and those over 80 years old still accounted for 1.95% (Figure 2B). We compared the age distribution pattern between the HPV-positive and HPV-negative groups of CSCC samples. The age distribution curves of the two groups were very similar, with no significant statistical differences (*p* > 0.05, Figure 2C). These findings suggested that HPV-negative CSCC should also be included in control measures to identify early detection methods and prevent misdiagnosis due to missed examination.

### 3.3. HPV genotype distribution in HPV-positive CSCC

In this study, HPV DNA genotype testing was performed using a Human Papillomavirus Genotyping kit for 23 Types (please see the Methods section for more details). Twenty-two different HPV genotypes were identified in CSCC tissues successfully, except HPV-83 genotype (Table 1). Among the 2,138 cases of HPV-positive CSCC, there were a total of 121 different combinations of HPV infection genotypes. Among them, there were 17 forms of single-type HPV infection, including all 17 HR-HPV genotypes, while 104 combinations of multiple-type HPV infections (data not shown). Our data showed that HR-HPV genotypes were not only presented in the form of single-type HPV infection in CSCC but also in the form of multiple-type mixed HPV infection. However, the five detected LR-HPV genotypes were only presented in multiple-type HPV mixed infection in CSCC tissues (Table 1).

**Table 1:** Genotype distribution of HPV infection in HPV-positive cervical squamous cell carcinoma.

HPV type	Genotype		Amount of single-type infection	Amount of multiple-type infection	Amount of two-type infection	Amount of three-type infections	Amount of four or more types infection	Amount of HPV-positive cases	Frequency of single-type infection	Frequency of multiple-type infection	HPV-positive rate
HR-HPV	1	HPV-16	1,324	225	184	31	10	1,549	71.61%	77.85%	72.45%
	2	HPV-18	133	67	53	11	3	200	7.19%	23.18%	9.35%
	3	HPV-58	96	62	45	13	4	158	5.19%	21.45%	7.39%
	4	HPV-52	55	50	36	8	6	105	2.97%	17.30%	4.91%
	5	HPV-31	69	33	25	6	2	102	3.73%	11.42%	4.77%
	6	HPV-33	53	39	29	6	4	92	2.87%	13.49%	4.30%
	7	HPV-59	37	20	10	8	2	57	2.00%	6.92%	2.67%
	8	HPV-45	20	14	9	2	3	34	1.08%	4.84%	1.59%
	9	HPV-51	7	12	6	5	1	19	0.38%	4.15%	0.89%
	10	HPV-56	7	12	9	1	2	19	0.38%	4.15%	0.89%
	11	HPV-73	8	11	7	2	2	19	0.43%	3.81%	0.89%
	12	HPV-66	9	9	4	1	4	18	0.49%	3.11%	0.84%
	13	HPV-39	11	5	4	1	0	16	0.59%	1.73%	0.75%
	14	HPV-53	5	11	8	3	0	16	0.27%	3.81%	0.75%
	15	HPV-68	4	10	7	2	1	14	0.22%	3.46%	0.65%
	16	HPV-82	7	3	1	2	0	10	0.38%	1.04%	0.47%
	17	HPV-35	4	4	2	2	0	8	0.22%	1.38%	0.37%
LR-HPV	18	HPV-11	0	25	12	7	6	25	0.00%	8.65%	1.17%
	19	HPV-6	0	20	11	5	4	20	0.00%	6.92%	0.94%
	20	HPV-81	0	8	3	3	2	8	0.00%	2.77%	0.37%
	21	HPV-42	0	7	7	0	0	7	0.00%	2.42%	0.33%
	22	HPV-43	0	7	4	1	2	7	0.00%	2.42%	0.33%
Total			1849	289	238	40	11	2,138	100.00%	100.00%	100.00%

The results indicated that the top eight high-frequency (greater than 1%) HR-HPV genotypes were HPV-16, -18, -58, -52, -31, -33, -59, and -45 in Chinese women with CSCC. Among them, HPV-16 was the most frequent one in both single-type and multiple-type HPV infection, accounting for 71.61% and 77.85%, respectively, followed by HPV-18 (7.19% and 23.18%), HPV-58 (5.19% and 21.45%) and HPV-52 (2.97% and 17.30%). Meanwhile, in the CSCC cases with LR-HPV positive, HPV-11 with multiple-type mixed infection had the highest frequency, accounting for 8.65% of cases (Table 1).

**Figure 3:**
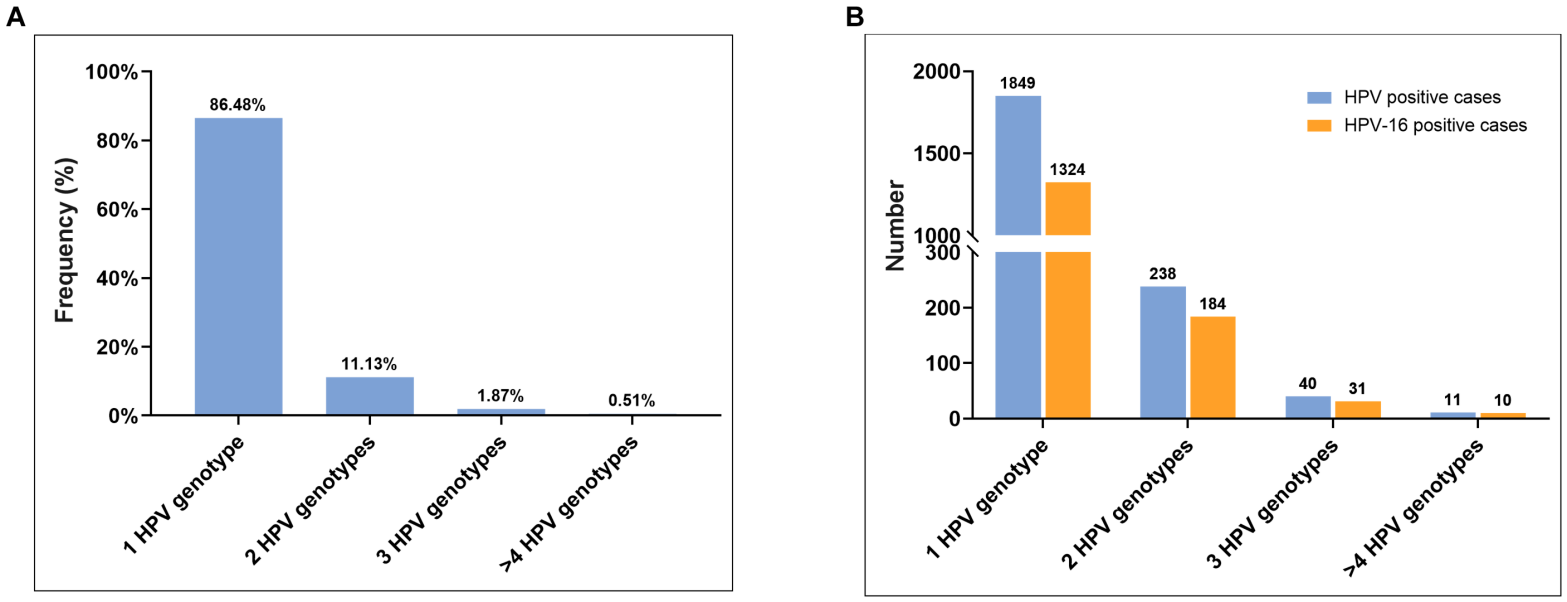
HPV genotype distribution in HPV-positive CSCC. **(A)** The prevalence of single-type HPV infection, two-type HPV mixed infection, three-type HPV mixed infection, and four or more types of HPV mixed infection. **(B)** The HPV-16 infection rate in different HPV infection form.

Of the 2,138 cases of HPV-positive CSCC, 86.48% (1849/2138) were found infected with single-type HPV, while 13.52% (289/2138) were identified with multiple-type HPV after de-redundancy. It should be noted that the cumulative total number of cases with multiple-types HPV infection in our study, would exceed 289 when calculating the frequency for each HPV genotype (Table 1). Specifically, 82.35% (238/289) of cases were infected with two types of HPV; 13.84% (40/289) of cases were infected with three types of HPV; while 3.81% (11/289) of cases were infected with four or more types of HPV (Figure 3A) with one case each infected with seven and nine types of HPV simultaneously.

Notably, among the 11 cases featuring four or more types of HPV infection, HPV-16 infection was detected in 90.9% (10/11) of cases. Meanwhile, HPV-16 infection was detected in 77.5% (31/40) of cases with three types of HPV mixed infection, 77.3% (184/238) of cases with two types of HPV mixed infection, and 71.6% (1,324/1849) of cases with single-type HPV infection (Figure 3B). Thus, our findings provide support for the assertion that HPV-16 is the most significant causative factor in the onset and progression of cervical cancer. Consequently, the timely detection and treatment of HPV-16 infection is crucial measure for preventing cervical cancer.

### 3.4. Attribution of common HR-HPV genotypes targeted by nine-valent HPV vaccine in cervical cancer

**Figure 4:**
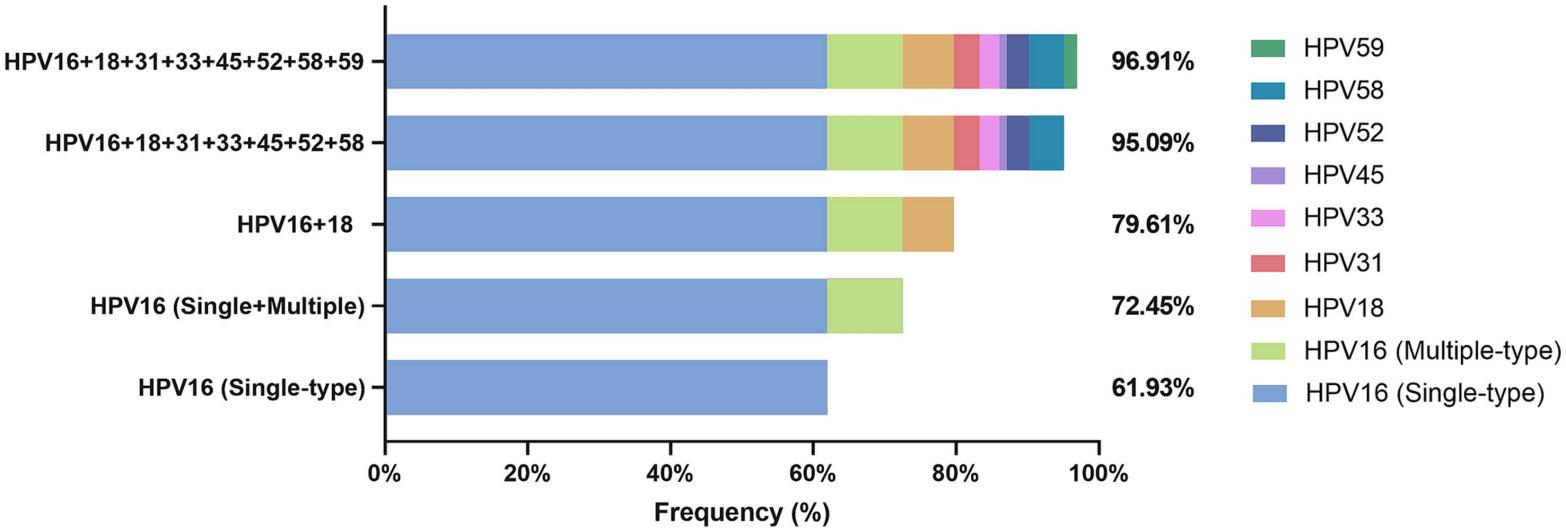
Cumulative attribution rates of common HR-HPV genotypes in HPV-positive CSCC cancer.

The HPV vaccine represents a significant preventative measure against HPV infection and serves as a crucial method for reducing the incidence of HPV-related cervical cancer. Among the HPV genotypes prevented by the nine-valent HPV vaccine, HPV-16, -18, -31, -33, -45, -52, and -58 are HR-HPV, while HPV-6 and HPV-11 are LR-HPV. In order to assess the efficacy of the nine-valent HPV vaccine in reducing the incidence of cervical cancer, we analyzed the cumulative attribution rates of the seven HR-HPV genotypes protected by the vaccine in CSCC with HPV infection. The results showed that the positive rate of single-type HPV-16 infection in CSCC was 61.93% (1,324/2,138); the cumulative positivity rate of HPV-16 single-type or multiple-type infection was 72.45% (1,549/2,138); while the cumulative positivity rate of both HPV-16 and HPV-18 infection was 79.61% (1,702/2,138). Additionally, the cumulative positivity rate of the seven HR-HPV types related to nine-valent HPV vaccine in CSCC reached 95.09% (2,033/2,138). Considering that HPV-59 is also a common HR-HPV genotype in central and eastern Chinese women (Table 1), we further calculated the cumulative positivity rate for the eight HR-HPV types (HPV16/18/31/33/45/52/58/59) in CSCC which was found to be 96.91% (2,072/2,138) (Figure 4). These findings suggest that effective prevention measures targeting these eight HPV types could potentially reduce the incidence of CSCC by approximately 97%.

### 3.5. Genotype distribution of HPV infection in various age groups in CSCC

**Figure 5:**
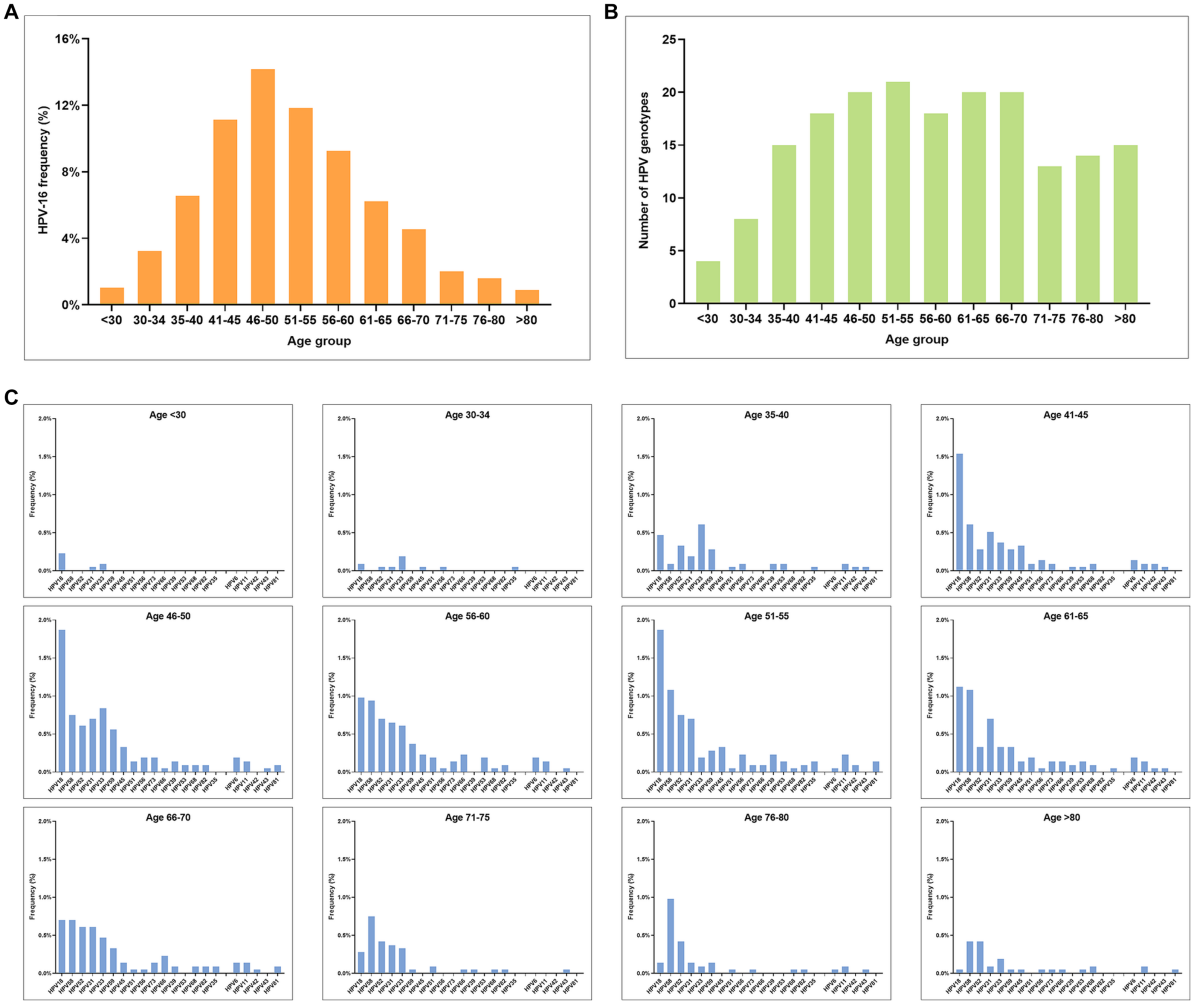
Prevalence of HPV infection in different age groups among HPV-positive CSCC. **(A)** Age distribution of HPV-16 infection. **(B)** Number of HPV infection genotypes in different age groups. **(C)** Genotype distribution and prevalence rate of HPV infection in different age groups (excluding HPV-16).

In this study, HPV-16 was identified as the most frequent detected HPV genotype across all age groups (Figure 5A). Notable, the peak of HPV infection was observed among HPV-positive CSCC cases aged 46–50 years old (Figure 5A), and the trend of HPV16 positivity rate was found to be similar to that of overall HPV infection (Figure 2B). In addition, the number of HPV infection genotypes in the eligible female population increases gradually with age (Figure 5B). At the same time, the positivity rate of each HPV genotype also increased gradually with age (Figure 5C). Among the age group, the highest prevalence and number of HPV infections was among women aged 40–60 years old, with a gradual decline observed in the group aged over 60 years. Notably, multiple HPV genotype infections were identified among individuals aged over 80 years (Figure 5C).

### 3.6. Comparison of the prevalence and genotype of HR-HPV between cervical cancer and healthy women undergoing cervical cancer screening

To compare the prevalence and genotype distribution of HR-HPV between cervical cancer patients and healthy individuals undergoing cervical cancer screening (screening population), we analyzed the HPV genotyping results of 10,245 individuals in the screening population in Jiangning District, Nanjing City, Jiangsu Province in 2022 (Table 2). Our results demonstrated a marked difference in the prevalence of common HR-HPV genotypes when comparing the screening population to cervical cancer patients. Specifically, the overall HPV positivity rate in screening population was 10.7% (1,097/10,245). Among them, the most common HPV genotypes were HPV-52 (2.25%), HPV-58 (1.60%), HPV-16 (1.31%), HPV-53 (1.22%), HPV-68 (0.93%), HPV-39 (0.92%), HPV-51 (0.78%), and HPV56 (0.74%). Among the eight common HR-HPV genotypes found in both cervical cancer patients and screening population, HPV-16 (ranked first in CSCC), HPV-58 (ranked third in CSCC), and HPV-52 (ranked fourth in CSCC) were found to be the same HPV genotypes. It was worth mentioning that the positivity rate of HPV-18 ranked only 14th in the screening population. Therefore, early detection and treatment of common HR-HPV genotypes associated with cervical cancer is critical for prevention and control of cervical cancer in China.

**Table 2:** Genotype distribution of HPV infection in healthy women undergoing cervical cancer screening.

HPV type	Genotype		Amount of single-type infection	Amount of two-type infection	Amount of three-type infections	Amount of four or more types infection	Amount of HPV-positive cases	Frequency in HPV-positive screening population	HPV-positive rate in screening population
HR-HPV	1	HPV-52	160	49	10	12	231	15.58%	2.25%
	2	HPV-58	105	39	11	9	164	11.06%	1.60%
	3	HPV-16	97	21	10	6	134	9.04%	1.31%
	4	HPV-53	63	40	16	6	125	8.43%	1.22%
	5	HPV-68	50	26	12	7	95	6.41%	0.93%
	6	HPV-39	53	22	12	7	94	6.34%	0.92%
	7	HPV-51	44	22	9	5	80	5.39%	0.78%
	8	HPV-56	39	18	13	6	76	5.12%	0.74%
	9	HPV-59	36	23	8	8	75	5.06%	0.73%
	10	HPV-33	37	19	9	3	68	4.59%	0.66%
	11	HPV-66	26	14	8	8	56	3.78%	0.55%
	12	HPV-35	20	12	5	7	44	2.97%	0.43%
	13	HPV-31	28	8	2	5	43	2.90%	0.42%
	14	HPV-18	23	10	2	4	39	2.63%	0.38%
	15	HPV-45	9	0	1	2	12	0.81%	0.12%
	16	HPV-73	4	2	1	0	7	0.47%	0.07%
	17	HPV-26	2	3	0	0	5	0.34%	0.05%
LR-HPV	18	HPV-81	7	17	9	5	38	2.56%	0.37%
	19	HPV-42	6	14	15	4	39	2.63%	0.38%
	20	HPV-43	1	12	6	4	23	1.55%	0.22%
	21	HPV-6	1	7	3	5	16	1.08%	0.16%
	22	HPV-40	1	7	3	4	15	1.01%	0.15%
	23	HPV-11	1	2	0	1	4	0.27%	0.04%
	Total		813	387	165	118	1,483	100.00%	/

## 4. Discussion

### 4.1. The infection rate of HR-HPV In CSCC patients is relatively high worldwide

HPV infection is closely associated the development of cervical cancer. The analysis of HPV infection rate is of great significance for developing prevention and control strategy of cervical cancer. A Meta-analysis of 5,081 cervical cancer found that the infection rates of HR-HPV were 90.6%, 87.8%, and 89.8% in North China, Northeast China, and East China, respectively (15). The prevalence of HPV infection was 95.9% of 339 CSCC patients in Hong Kong, China (16). Another meta-analysis of 79 studies with 5,954 Asian cervical cancer patients found the HPV infection rate to be 85.9% (17). De Sanjose et al. conducted a retrospective analysis on 10,575 cervical cancer patients worldwide and found that 85% were HPV-positive in their paraffin tissue (18). The HPV infection rates were 88%, 87%, 91%, 82%, 95%, and 79% in Asia, Europe, North America, South America, Oceania, and Africa, respectively (18). In our study, the infection rate of HPV in CSCC patients was 92.71%, which was higher than the rates reported worldwide. The findings highlighted the urgency of preventing and controlling HPV-associated cervical cancer.

### 4.2. Women over 65 years old should be included in cervical cancer screening in China

Our study showed that the highest incidence of cervical cancer was observed in individuals aged 40–60 years, accounting for 61.57% of all CSCC cases. Of note, the incidence rate in the group over 66 years old was still 16.35%. Among them, the incidence rate of cervical cancer was 11.54% in the women aged 66–75 years old, 2.86% in those aged 76–80 years old, and 1.95% in those over 80 years old (Figure 2B).

Our data indicated that the HPV-16 infection rate was consistently high across all age groups of cervical cancer, accounting for 72.45% of CSCC cases (Figure 5A, Table 1). Previous study also found that HPV-16 was the most common HR-HPV type (72%) and the most common persistence infection type (16%) in older adult women (19). Moreover, with increasing age, there was a gradual increase in the number of mixed infections with different HPV genotypes (Figure 5B), as well as the frequency of infection with each HPV genotype (Figure 5C). The peak of HPV infection is reached at 40–60 years old, and gradually decreases after 60 years old. Multiple HPV infections are still present in cases over 80 years old (Figure 5C). Zhao reported the prevalence of HPV infection in older adult women (≥60 years old) and the significance in older adult cervical lesions (20). Their results showed that the overall infection rate of HR-HPV was 16.73% (84/502), which was consistent with our results in the group aged over 65 years (16.3%). Their results also showed that of 39 cases who had underwent cervical biopsy by colposcopy, 5 cases were diagnosed with cervical cancer (5.62%), 10 cases were CINII+ (11.24%), and 5 cases were CINIII (5.62%). Previous studies showed HR-HPV prevalence of older adult women in Guangxi are higher than those of middle-aged women and caused higher CIN incidence rate (21, 22). Although the HPV infection rate decreases in older adult patients compared with young women, the pathogenicity of HPV does not decrease (20).

So, it should be given sufficient attention to older adult patients with HPV-positive infection. In addition, with the rapid development of China’s economy, the average life expectancy of the Chinese population has significantly increased. A recent study indicated that life expectancy in China is expected to increase steadily from 77.7 years in 2019 to 79 years by 2030 and 81.3 years by 2035, with an average of 85.1 years for women and 78.1 years for men (23). In our study, the number of cervical cancer patients aged 66–80 years old represented 14.40% of all CSCC cases. Therefore, it is necessary to include the older adult population aged 66–80 years old in the cervical cancer screening and control strategy in China.

### 4.3. The order of HPV genotype prevalence in cervical cancer tissues varies between China and other countries

In this study, the total HPV detection rate was 92.71% (2,138/2306) in 2,306 CSCC tissues, including 86.48% (1849/2138) for single type HPV detection rate and 13.52% (289/2138) for multiple-type detection rate. Among the cases with multiple genotypes infection, 238 cases (11.13%) had two HPV genotypes infection, 40 cases (1.87%) had three HPV genotypes infection, and only 11 cases (0.51%) had four or more HPV genotypes infection (Figure 3B). Additionally, our results suggest that LR-HPV genotypes (such as HPV-6, -11, -42, -43, -81, and -83) are rare to detect in CSCC tissues. When these LR-HPV genotypes are detected, they are often found in mixed infection with other HR-HPV genotypes. Nevertheless, HR-HPV genotypes, including HPV-16, -18, -31, -33, -35, -39, -45, -51, -52, -56, -58, and -59, are closely related to the development of CSCC, with HPV-16 and HPV-18 being the most common types in CSCC tissues.

The top eight HPV genotypes of cervical cancers in Chinese women are HPV-16, -18, -58, -52, -31, -33, -59, and -45, which differ from the commonly occurring HR-HPV genotypes in other countries. Literature reports suggested that HPV-35 and HPV-45 were common in cervical cancer tissues in West Africa (5, 24). Our data show that the positivity rate of HPV-59 was the seventh of the top eight HR-HPV genotypes in China, while HPV-35 has the lowest positivity rate (0.37%) among the commonly occurring HR-HPV genotypes in China (Table 1). This may be related to differences in HPV detection techniques or HPV genotyping profiles, as well as differences in specimen types and quantities, regions, and ethnicities. de Sanjose reported 85% (8,977/10,575) cases had positive results of HPV DNA (18). Among these cases, 8,977 (85%) had positive results of HPV DNA. The most common HPV types were HPV-16, -18, -31, -33, -35, -45, -52, and -58, with a total global relative contribution rate of 8,196 (91%) of the 8,977 cases (18). This study shows that there’s variability in the risk of cervical cancer due to different HR-HPV genotypes, and it is significant to study the distribution patterns of different HR-HPV genotypes in cervical cancer tissues (18). The WHO recommends that each country should establish its own HPV genotyping database for cervical carcinoma, which will be an important guide for the prevention and treatment of CSCC and the development of prophylactic HPV vaccines (25, 26).

Our study also found that the positivity rate of HPV-16 and HPV-18 were the highest among all genotypes detected in CSCC tissues, with a percentage of 79.61% for both genotypes, while HR-HPV genotypes, HPV-31 and HPV-33 ranked as the fifth and sixth most prevalent genotypes (9.07% for both genotypes), which is consistent with the low carcinogenic risk of them. In China, it is important to pay attention to the HR-HPV genotypes HPV-52 and HPV-58 regional ranked third and fourth regionally (detection frequency of both was 12.3%), as well as HPV-59 and HPV-45 (the detection frequency was 2.67% and 1.59%, respectively). Therefore, HR-HPV genotyping testing for cervical cancer in women is an important clinical reference index of the need for colposcopy and close follow-up in women over 30 years old.

### 4.4. Rational HPV genotyping profile can improve the efficiency and effectiveness of cervical cancer screening

In our study, the overall incidence rates of HR-HPV and LR-HPV in central and eastern China were 13.16% and 1.32% in cervical cancer screening individuals, respectively (Table 2). Of which the common HPV genotypes in healthy women were HPV-52, -58, -16, -53, -68, -39, -51, and -56, with positive rates of 2.25%, 1.60%, 1.31%, 1.22%, 0.93%, 0.92%, 0.78%, and 0.74%, respectively (Table 2). Our results also identified that the cases of HPV infection genotype and frequency were significantly lower in people under 30 than in other age groups, which is supported by the study of Fatima Mijit et al. (27). This suggests that the lower positivity rate of HPV in women under 30 may be due to their higher immune levels (28, 29). However, cases of advanced cervical cancer have also been reported in women under 30 years old in recent years (30). Our results showed that there were 41 cases of CSCC in women under 30 years old, with the youngest diagnosis for a 19-year-old patient (Figure 2B). Therefore, it is an important way to prevent cervical cancer, through taking personal protective measures, reducing the chances of HPV infection, and improving immunity.

On July 6, 2021, the World Health Organization (WHO) and the United Nations Population Fund (UNFPA) released the second edition of the “Cervical Cancer Screening and Treatment Guidelines for Precancerous Lesions,” which clearly recommended HPV-DNA testing as the preferred screening method for cervical cancer over the current cytology or Pap smear (25). HPV DNA testing is more cost effective in terms of sensitivity and specificity of the test technology. Currently, many companies tend to develop HPV testing kits that can detect as many HPV genotypes as possible (31). However, excessive HPV genotypes will inevitably increase the complexity of multiple PCR technologies and the cost of reagents, resulting in high terminal price and increased difficulty in promotion. This study found that the most common HR-HPV genotypes in Chinese CSCC were HPV-16, -18, -58, -52, -31, -33, -59, and -45, with positive rates of 72.45%, 9.35%, 7.39%, 4.91%, 4.77%, 4.30%, 2.67%, and 1.59%, respectively. These eight HR-HPV genotypes can be detected in 96.93% of CSCC cases (Figure 4). The detection rate of the other nine HPV types was only 3.07%. Considering that the WHO recommends HPV DNA testing as the preferred screening method for cervical cancer (25), and based on our results, we suggest that the eight common high-frequency HPV genotypes, HPV-16, -18, -58, -52, -31, -33, -59, and -45 in China, should be prioritized as targets for multiple nucleic acid testing to reduce technical complexity and reagent costs and lay the foundation for community or home-based promotion.

Our current findings suggest that including the L1 protein of HPV-59 in the protective profile of HPV prophylactic vaccine could improve the protection rate of HPV vaccines. However, it is important to note that this conclusion should be further confirmed by large size real-world studies.

We also strongly recommend that Chinese women should receive polyvalent (such as nine-valent) vaccines whenever possible, which can achieve maximum vaccine protection through one vaccination program (32).

In conclusion, HPV is a common sexually transmitted infection worldwide that disrupts normal social life and has deadly consequences. With few exceptions, the burden of HPV infection and related diseases remains high in developing countries. Factors contributing to these high rates include poor living conditions, co-infection with other pathogens, poor health care facilities, and the high cost of vaccines. China is one of the countries with a high incidence of HPV infection and cervical cancer. Therefore, it is of great significance to study HPV infection patterns in patients with cervical squamous cell carcinoma and healthy individuals, and to develop new vaccines against HPV and new technologies for the rapid detection of HPV based on the high incidence of HPV genotypes in the Chinese population. This will accelerate the elimination of cervical cancer worldwide.

## Data availability statement

The original contributions presented in the study are included in the article/Supplementary material, further inquiries can be directed to the corresponding authors.

## Ethics statement

The studies involving humans were approved by the Ethics Committee of Nanjing Traditional Chinese Medicine Hospital. The studies were conducted in accordance with the local legislation and institutional requirements. The participants provided their written informed consent to participate in this study.

## Author contributions

JG, ZW, and JH were responsible for study concept and design. CH, MY, JiL, JC, JJ, HW, LX, JuL, DL, JG, ZW, and JH were responsible for specimens sampling. CH, WH, and JG were responsible for the experiments in this study. JG, WH, RZ, and JH were responsible for the acquisition, analysis, and interpretation of data. WH, RZ, and JH were responsible for drafting the manuscript. All authors contributed to the article and approved the submitted version.

## Funding

This study was supported by grants from Science and Technology Innovation Action Plan Startup Project (Sail Special) of Shanghai (grant number: 22YF1420500), the Fundamental Research Funds for the Central Universities (grant number: KLSB2022QN-01), Medical-Industrial Crossover Research Fund of Shanghai Jiao Tong University (grant numbers: YG2022QN070 and 19X190020005), and the National Natural Science Foundation of China (grant number: 81872274).

## Conflict of interest

The authors declare that the research was conducted in the absence of any commercial or financial relationships that could be construed as a potential conflict of interest.

## Publisher’s note

All claims expressed in this article are solely those of the authors and do not necessarily represent those of their affiliated organizations, or those of the publisher, the editors and the reviewers. Any product that may be evaluated in this article, or claim that may be made by its manufacturer, is not guaranteed or endorsed by the publisher.
