# Supplementary material for: HPV prevalence and genotype distribution in 2,306 patients with cervical squamous cell carcinoma in central and eastern China
Source: Front Public Health. 2023 Aug 28;11:1225652. doi: 10.3389/fpubh.2023.1225652 (PMC10493278; doi:10.3389/fpubh.2023.1225652)
Supplement: Supplementary file 1 [file Table_1.DOCX]

Supplementary Material

HPV prevalence and genotype distribution in 2,306 patients with cervical squamous cell carcinoma in central and eastern China

**Chunrong Han^1†^, Wanqiu Huang^2†^, Mei Ye^3†^, Rong Zou^2†^, Jianyun Lan^4^, Jing Chen^5^, Jingui Jiang^6^, Hongjing Wang^7^, Lin Xia^8^, Jun Luo^9^, Dongbin Li^10^, Jianxiang Geng^11,13*^, Zhihui Wang^12,13*^ and Jian Huang^2,13*^**

^1^Department of Pathology, Nanjing Lishui People's Hospital (Zhongda Hospital Lishui Branch), Southeast University, Nanjing, China.

^2^Key Laboratory of Systems Biomedicine (Ministry of Education), Shanghai Centre for Systems Biomedicine, Shanghai Jiao Tong University, Shanghai, China.

^3^Nanjing Jiangning District Maternal and Child Health and Family Planning Service Center, Nanjing, China.

^4^Department of Pathology, Yancheng No.1 People’s Hospital, Yancheng, China.

^5^Department of Pathology, Jingjiang People's Hospital, Taizhou, China.

^6^Department of Pathology, Jinhu County People's Hospital, Huai’an, China.

^7^Department of Pathology, Dantu District People's Hospital of Zhenjiang, Zhenjiang, China.

^8^Department of Pathology, People's Hospital of Yangzhong City, Yangzhong, China.

^9^Department of Central Laboratory, Jiangsu Health Vocational College, Nanjing, China.

^10^Department of Pathology, Nanjing Meishan Hospital, Nanjing, China.

^11^Department of Pathology, Nanjing Hospital of Traditional Chinese Medicine, Nanjing, China.

^12^Department of Pathology, Linyi Cancer Hospital, Linyi, China.

^13^The Cross-Strait Precision Medicine Association HPV Infection Disease Professional Committee, Nanjing, China.

**^†^** These authors have contributed equally to this work and share first authorship.

*** Correspondence:**Jian Huang: jianhuang@sjtu.edu.cn
Zhihui Wang: young0831@sina.com
Jianxiang Geng: njgjx2022@163.com

**Supplementary Table 1. The list of the details for 48 hospitals in this study.**

| **No.** | **Name of Hospitals** | **City** | **Province** |
| --- | --- | --- | --- |
| 1 | Anqing First People's Hospital of Anhui Province | Anqing | Anhui |
| 2 | Dangtu County People's Hospital of Anhui Province | Ma'anshan | Anhui |
| 3 | 17th Metallurgical Hospital of Ma'anshan City | Ma'anshan | Anhui |
| 4 | Central Hospital of Ma'anshan City | Ma'anshan | Anhui |
| 5 | Shangcheng County People's Hospital of Henan Province | Xinyang | Henan |
| 6 | Changshu First People's Hospital of Jiangsu Province | Suzhou | Jiangsu |
| 7 | Changshu Traditional Chinese Medicine Hospital of Jiangsu Province | Suzhou | Jiangsu |
| 8 | Dafeng People's Hospital of Jiangsu Province | Yancheng | Jiangsu |
| 9 | Jinhu County People's Hospital of Huai'an City | Huai'an | Jiangsu |
| 10 | Jiangsu University Affiliated Hospital of Zhenjiang City | Zhenjiang | Jiangsu |
| 11 | Jinhu Maternal and Child Health Hospital of Jiangsu Province | Jinhu Mat | Jiangsu |
| 12 | Jingjiang People's Hospital of Jiangsu Province | Huan'an | Jiangsu |
| 13 | Kunshan First People's Hospital of Jiangsu Province | Suzhou | Jiangsu |
| 14 | Dachang Hospital of Nanjing City | Nanjing | Jiangsu |
| 15 | Nanjing Maternal and Child Health Hospital | Nanjing | Jiangsu |
| 16 | Gaochun District People's Hospital of Nanjing City | Nanjing | Jiangsu |
| 17 | Jiangning Hospital of Nanjing City | Nanjing | Jiangsu |
| 18 | Lishui District People's Hospital of Nanjing City | Nanjing | Jiangsu |
| 19 | Liuhe District People's Hospital of Nanjing City | Nanjing | Jiangsu |
| 20 | Meishan Hospital of Nanjing City | Nanjing | Jiangsu |
| 21 | Mingji Hospital of Nanjing City | Nanjing | Jiangsu |
| 22 | Tongren Hospital of Nanjing City | Nanjing | Jiangsu |
| 23 | Integrated Traditional Chinese and Western Medicine Hospital of Nanjing City | Nanjing | Jiangsu |
| 24 | Nanjing Traditional Chinese Medicine Hospital | Nanjing | Jiangsu |
| 25 | Peixian People's Hospital of Jiangsu Province | Xuzhou | Jiangsu |
| 26 | Pizhou People's Hospital of Jiangsu Province | Xuzhou | Jiangsu |
| 27 | Qidong People's Hospital of Jiangsu Province | Nantong | Jiangsu |
| 28 | Sheyang County People's Hospital of Jiangsu Province | Yancheng | Jiangsu |
| 29 | Shuyang County Traditional Chinese Medicine Hospital of Jiangsu Province | Suqian | Jiangsu |
| 30 | Changshu Traditional Chinese Medicine Hospital of Suzhou City | Suzhou | Jiangsu |
| 31 | Ninth People's Hospital of Suzhou City | Suzhou | Jiangsu |
| 32 | Suzhou Municipal Hospital | Suzhou | Jiangsu |
| 33 | Xishan People's Hospital of Wuxi City | Wuxi | Jiangsu |
| 34 | Xinghua People's Hospital of Jiangsu Province | Taizhou | Jiangsu |
| 35 | Maternal and Child Health Hospital of Xuzhou City | Xuzhou | Jiangsu |
| 36 | Xuzhou Traditional Chinese Medicine Hospital | Xuzhou | Jiangsu |
| 37 | First People's Hospital of Yancheng City | Yancheng | Jiangsu |
| 38 | Yangzhong People's Hospital of Jiangsu Province | Zhenjiang | Jiangsu |
| 39 | Hongquan Hospital of Yangzhou City | Yangzhong | Jiangsu |
| 40 | Jiangdu People's Hospital of Yangzhou City | Yangzhong | Jiangsu |
| 41 | First People's Hospital of Zhangjiagang City | Zhangjiagang | Jiangsu |
| 42 | Dantu District People's Hospital of Zhenjiang City | Zhenjiang | Jiangsu |
| 43 | First Affiliated Hospital of Soochow University | Suzhou | Jiangsu |
| 44 | First People's Hospital of Jiujiang City in Jiangxi Province | Jiujiang | Jiangxi |
| 45 | Linyi Tumor Hospital of Shandong Province | Linyi | Shandong |
| 46 | Leqing Maternal and Child Health Hospital of Zhejiang Province | Wenzhou | Zhejiang |
| 47 | Yinzhou People's Hospital of Ningbo City | Ningbo | Zhejiang |
| 48 | First Affiliated Hospital of Wenzhou Medical University | Wenzhou | Zhejiang |
